# Body fluids may contribute to human-to-human transmission of severe acute respiratory syndrome coronavirus 2: evidence and practical experience

**DOI:** 10.1186/s13020-020-00337-7

**Published:** 2020-06-05

**Authors:** Amir Hossein Mohseni, Sedigheh Taghinezhad-S, Zhigang Xu, Xiangsheng Fu

**Affiliations:** 1 Digestive Endoscopy Center, The Affiliated Hospital of North Sichuan Medical College, Road Wenhua 63#, Region Shunqing, Nanchong, Sichuan 637000 China; 2 Affiliated Hospital of Traditional Chinese Medicine of Southwest Medical University Luzhou, Luzhou, 646000 China; 3 Red Cross Hospital of Wuhan City, Wuhan, 430015 China

**Keywords:** Coronavirus, SARS-CoV-2, Transmission, COVID-19, Prevention

## Abstract

**Background:**

In December 2019, an unbelievable outbreak of pneumonia associated with coronavirus was reported in the city of Wuhan, Hubei Province. This virus was called severe acute respiratory syndrome coronavirus 2 (SARS-CoV-2). Although much effort has been spent on clarifying the transmission route of SARS-CoV-2, but, very little evidence is available regarding the relationship between human body fluids and transmission of SARS-CoV-2 virus. Considerable evidence from hospital in Wuhan indicates that strict rules to avoid occupational exposure to patients’ body fluids in healthcare settings, particularly among every medical staff, limited person-to-person transmission of nosocomial infections by direct or indirect contact.

**Conclusion:**

We tried to provide important information for understanding the possible transmission routes of SARS-CoV-2 via body fluids including bronchoalveolar-lavage, saliva, blood, urine, feces, sputum, tears, and semen in order to control coronavirus disease 2019 (COVID-19) occurrences.

## Background

The severe acute respiratory syndrome coronavirus 2 (SARS-CoV-2) showed a rapid spread around the world and, for this reason, infection due to the SARS-CoV-2 virus as coronavirus disease 2019 (COVID-19) was officially declared as a pandemic by the WHO on March 12, 2020. Despite continuous endeavors by scientists, to date little progress has been made to create a specific medicine and effective vaccine against COVID-19. Previous studies have found the effective impact of traditional Chinese herbal medicine on treatment of SARS and improvement of lung damage caused by influenza viruses, thus showing its role as a more immediate treatment of COVID-19, but its exact mechanism of action remains unclear [1]. Correspondingly, in silico analysis emphasized the potential anti-SARS-CoV-2 activity of traditional Chinese herbal medicine in treating viral respiratory infections [2]. Therefore, more attention must also be paid to use traditional Chinese medicine as one of the most promising approaches in battling the outbreak.

Based on the guideline of WHO, droplets and fomites are generally considered the most important transmission factors of SARS-CoV-2. Despite that, the data related to the transmission routes do not always meet the aforementioned standards. Therefore, this work aims to provide an overview of the different human-to-human transmission routes of SARS-CoV-2 through infected body fluids in an attempt to control COVID-19 outbreaks.

### Transmission routes of SARS-CoV-2

The ability of the SARS-CoV-2 to enter the cell is the crucial factor characterizing the infection. In this respect, is a well-known receptor for binding of SARS-CoV-2 binds to the angiotensin-converting enzyme 2 (ACE2) in human cells through its spike protein. Recently, some studies proposed a route to explain how SARS-CoV-2 can infect the gastrointestinal tract [3], since some SARS-CoV-2 patients showed gastrointestinal symptoms and this virus was also isolated from feces and anal swabs performed on these patients [4]. Indeed, SARS-CoV-2 was recently detected in the urine, tears and conjunctival secretions in SARS-CoV-2 patients [5, 6]. Several lines of evidence suggested that the vagina can be infected by HCoV-229E, confirming the role of the sexual route in the transmission of the HCoV-229E virus [7]. Unexpectedly, a more recent study by Cui et al. found that ACE2 receptor is not expressed in the vagina and cervix tissues. They pointed out that the pregnant SARS-CoV-2 patients do not have SARS-CoV-2 in their vaginal discharge hence, SARS-CoV-2 cannot be transmitted during the delivery [8]. More specifically, SARS-CoV-2 is absent in the cord blood, neonatal throat swab, breastmilk and amniotic fluid [9]. Although this evidence confirmed the impossibility of vertical transmission of the SARS-CoV-2 virus, a recent study published by another group showed that the fetal liver is an organ that can be infected by SARS-CoV-2 during pregnancy because of the up-regulation of the ACE2 receptor expression [10].

Considering that the genome of the SARS-CoV-2 is more than 89% similar to the SARS-like coronaviruses group, we can estimate that the transmission behavior is similar in these two viruses [11]. Because of the presence of the ACE2 receptor in some organs such as lung, gastrointestinal tract, liver, heart, kidneys, testis, and placenta, we believe that the transmission of SARS-CoV-2 is not limited to the respiratory transmission. It is thus predictable that the exposure to human body fluids such as bronchoalveolar-lavage, saliva, blood, urine, feces, sputum, tears, and semen especially among asymptomatic patients may represent a risk factor for the invasion of the virus into the human body. To confirm this hypothesis, previous studies regarding SARS-CoV described the potential route of contamination via sweat gland cells in the skin, suggesting its possible role in the transmission of SARS-CoV-2 to individuals who are in an unprotected contact or in direct contact with the skin of infected patients [12]. In addition, the transmission of SARS-CoV-2 via semen is potentially possible due to the presence of ACE2 in the cells of the testis. However, this specific type of transmission still remains unclear.

### Practical experience in preventing the transmission of SARS-CoV-2

**Fig. 1:**
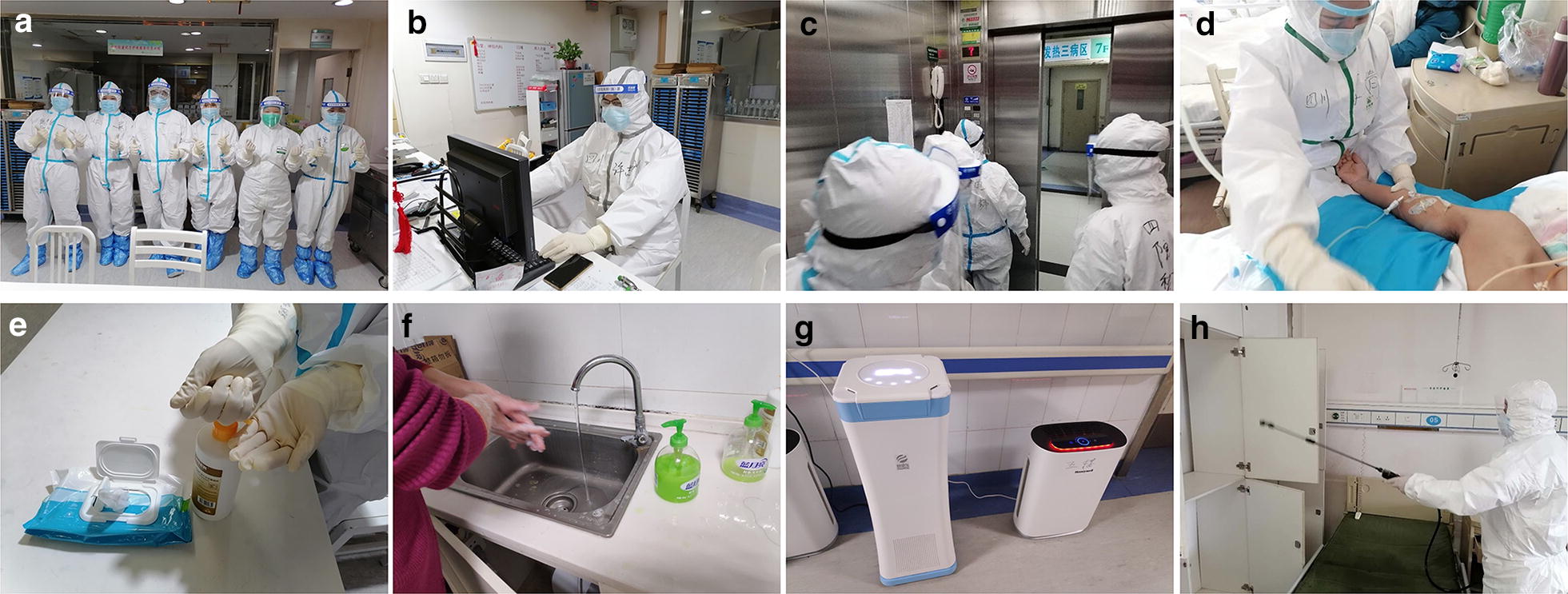
Practices and experiences to prevent nosocomial infections in the designated hospitals of Wuhan because of the presence of patients infected with COVID-19. **a** Medical staff wearing and taking off disposable medical protective clothing according to the strict procedures; **b** all medical staff is in full compliance with the strict rules to prevent nosocomial infections during work; **c** medical staff entering contaminated zone according to the strict procedures; **d** wear latex gloves when touching the patient; **e** disinfect the gloves before and after touching the patients; **f** wash your hands before and after meals and defecation; **g** purify and disinfect the air in the offices and wards; **h** disinfect the wards after patients leaving

Fortunately, until recently, China is successful in bringing the epidemic under control. On March 7, all the mobile cabin hospitals in Wuhan discharged the patients. Most remarkably, according to the official news in China on March 6, no COVID-19 infection was reported among 42,000 doctors and nurses who rushed from other provinces of China to the rescue of Hubei Province, many of them working there for more than 2 months. Chinese results against SARS-CoV-2 in the present stage may be attributed to the fact that the hospitals in Wuhan formulated strict rules to prevent nosocomial infections, and every medical staff fully followed these rules. The core rule is to avoid direct touch of body fluid of patients, including feces, urine and nasal mucus, and avoid direct physical contact with patients and among each other. For example, the medical staff must wear and take off disposable medical protective clothing according to the strict procedures during working, and must wear latex gloves when touching the patient or the blood/fluid of patients. During procedures on the airways, in which aerosol or splash may occur, the staff must wear goggles or protective screens, disposable impermeable protective clothing, and even a respirator if necessary. Patients must wear surgical masks and keep a safe distance with each other, and wash hands before and after meals and defecation. The wards and offices were frequently cleaned and disinfected. Object surfaces were also cleaned and disinfected, including medical instruments, elevator buttons, door handles, computer keyboards and mouse (Fig. 1).

## Conclusion

Usually, viruses with pandemic potential like SARS-CoV-2 can survive for a long time on dry surfaces, resulting in a broad level of contamination in the environment. Therefore, improving the human behaviors to overcome the outbreak is urgently needed. We can speculate that human body fluids from organs expressing the ACE2 receptor may be infected with SARS-CoV-2 virus. Therefore, strict precautions should be taken into consideration during the handling of the excretions of SARS-CoV-2 patients and the sewage of hospitals to prevent COVID-19 infection. Finally, wearing appropriate personal protective equipment, and performing strict and frequent hand hygiene should be taken into account. Overall, well designed inter-human SARS-CoV-2 transmission studies concerning human body fluids can fill the knowledge gap and help to determine the risk assessment regarding different transmission routes, resulting in a fast and global management of the outbreak.

## Data Availability

Not applicable.

## Abbreviations

SARS-CoV-2: Severe acute respiratory syndrome coronavirus 2
COVID-19: Coronavirus disease 2019
ACE2: Angiotensin-converting enzyme 2
